# Case Report: Repeated Intralesional Injections of Autologous Mesenchymal Stem Cells Combined With Platelet-Rich Plasma for Superficial Digital Flexor Tendon Healing in a Show Jumping Horse

**DOI:** 10.3389/fvets.2022.843131

**Published:** 2022-02-18

**Authors:** Luca Melotti, Anna Carolo, Noha Elshazly, Filippo Boesso, Laura Da Dalt, Gianfranco Gabai, Anna Perazzi, Ilaria Iacopetti, Marco Patruno

**Affiliations:** ^1^Department of Comparative Biomedicine and Food Science, University of Padua–Agripolis Campus, Legnaro, Italy; ^2^Tissue Engineering Laboratories, Faculty of Dentistry, Alexandria University, Alexandria, Egypt; ^3^Veterinary Practicioner, Padua, Italy; ^4^Department of Animal Medicine, Production and Health, University of Padua–Agripolis Campus, Legnaro, Italy

**Keywords:** SDFT, platelet-rich plasma, mesenchymal stem cells, regenerative medicine, equine orthopedics, tissue regeneration, horse

## Abstract

In the present case report a show jumping 10-year-old Sella Italiano gelding, presented with severe lameness, swelling and pain at palpation of the mid-metacarpal region of the left forelimb. Clinical and ultrasound examination diagnosed a chronic tendonitis of the central region of the superficial digital flexor tendon (SDFT). The lesion was a reoccurrence since it developed from a previously healed injury. The horse had to stop competing and was unresponsive to gold-standard treatments as Non-steroidal anti-inflammatory drugs (NSAIDs) and conservative management after 6 months of therapy. The animal was subjected to repeated intralesional injections of autologous adipose-derived mesenchymal stem cells (AD-MSCs) combined with autologous platelet-rich plasma (PRP). The combined treatment was administered twice in a 1-month interval. The healing process was assessed through clinical examination, ultrasound imaging and quantification of oxidative stress products and inflammatory mediators in blood plasma. After 2 weeks from first injection, a reduction of concentration of oxidative-derived products was observed, together with an increase of anti-inflammatory cytokines and pro-mitotic growth factors. These results were reflected clinically as the horse showed a reduction of lameness along with swelling and pain after 4 weeks. At the 1-year follow-up, the horse showed no signs of lameness and swelling. The ultrasonographic examination highlighted a compact fiber alignment with a normal echogenic tendon as observed in the sound contralateral limb. Moreover, the horse went back to the previous level of competition. Our results suggest the positive effects of a repeated intralesional injection of AD-MSCs and PRP for the treatment of a chronic tendonitis with long-term effects and an improvement for both equine quality of life and athletic performance.

## Introduction

Tendinopathies are one of the most common orthopedic disorders in equine and human athletes, leading to lameness and pain (1–5). Tendon injuries are responsible for approximately one third of traumas that occur during the sporting career of horses (6, 7), forcing a significant number of individuals to an early retirement from competition (8, 9). The superficial digital flexor tendon (SDFT) is frequently injured in show jumping discipline due to the repeated and excessive loading forces that the tendon has to sustain after jumping and landing (10, 11). Tendonitis affecting the SDFT have an incidence up to 43%, and most of them occur in the central tendon region (6, 12).

Tendons possess a limited regenerative capacity and usually heal by forming a fibrotic scar, but the repaired tissue possesses inferior biomechanical characteristics compared to its normal physiological counterpart (13–15). Consequently, horses that have previously sustained a tendon injury are more prone to re-injury (up to 80%) or to chronicity (9, 16).

The gold-standard treatments for tendon injuries consist of conservative therapies including administration of non-steroidal anti-inflammatory drugs (NSAIDs) and rehabilitation aiming to attenuate symptoms and to recover tendon function. Although clinical improvements might be observed (i.e., relief of symptoms), most of these options lack long-term therapeutic success (17, 18). Over the last two decades, regenerative therapies have been gaining interest because of their beneficial effects in supporting and stimulating the healing process, leading to a healed tissue that resembles healthy tendon in structure and function (19, 20).

Mesenchymal stem cells (MSCs) derived from multiple sources, as bone marrow (BM-MSCs), adipose tissue (AD-MSCs), or peripheral blood (PB-MSCs), have proved their efficacy in improving tendon healing in horses thus reducing the reoccurrence rate of injury, mainly because of their paracrine activity (21–24). Different route of administration of MSCs, including intralesional injection, have demonstrated to be a safe and effective practice to treat tendon injury in equine medicine (25, 26). Another product of interest in equine regenerative practice is platelet-rich plasma (PRP), a blood-derived product rich in growth factors and cytokines that can sustain and boost the tissue healing process (20, 27, 28). When combined, these two treatments possess a higher regenerative potential in comparison to their application alone as it has been demonstrated for treating different tissue (e.g., skin, bone, joint) in human and horses (9, 29–33). Nevertheless, only one study describes the repeated application of MSCs and PRP for the treatment of naturally occurring chronic tendonitis, which was not a re-injury, in the equine in a 16-weeks time interval (34).

In terms of risk factors (e.g., age and over-exercise) and etiology, human and horses share a similar pathophysiology of tendinopathies. For this reason, studies to test regenerative therapies in the horse including cell and cell-free treatments, or their combination, for tendon healing might be useful as preclinical data for translation purposes to human medicine (35–38).

In this case report, we describe the repeated application of autologous adipose-derived MSCs and autologous PRP for the treatment of a chronic recurrent SDFT tendonitis developed from a previous injury in a show jumping horse.

## Case Description

### Clinical History

A 10-year-old Sella Italiano gelding, competing in show jumping, presented with a lesion in the SDFT of the left forelimb in the middle third of the metacarpal region. The lesion was a reoccurrence, which had developed from a previous healed injury in the same area of the SDFT. At diagnosis, the horse showed a lameness grade 2.5/5 based on the American Association of Equine Practioners (AAEP) scale (as reported in Table 1). Pain and local heat were noted at palpation along with severe swelling.

**Table 1:** Clinical and ultrasonographic scores to assess lameness, echogenicity and fiber alignment.

**Score**	**AAEP degree of lameness**	**Echogenicity**	**Fiber alignment (FA)**
0	Lameness not perceptible under any circumstances	Normal echogenicity	≥75% parallel fiber bundles in the lesion
1	Lameness is difficult to observe and is not consistently apparent, regardless of circumstances	Mildly hypoechoic	50–74% parallel fiber bundles in the lesion
2	Lameness is difficult to observe at walk or when trotting in a straight line, but consistently apparent under certain circumstances	Moderate hypoechoicity	25–49% parallel fiber bundles in the lesion
3	Lameness is consistently observable at a trot under all circumstances	Severe hypoechoicity	≤25% parallel fiber bundles in the lesion
4	Lameness is obvious at walk	–	–
5	Lameness produces minimal weight bearing in motion and/or at rest or a complete inability to move	–	–

Six months ago, when diagnosed, the horse stopped competing and was treated with NSAIDs. Furthermore, a controlled rehabilitation exercise program was followed, adapted and based on the type of injury. The program started with a complete stall rest for the first 2 weeks. Then it was followed by a gradual increase of walking and trotting exercises, starting with 5 min walking a day with an increase of 5 min every 2 weeks, up to 40 min. After 20 weeks, the horse began to trot for 2 min each day with a progressive increase of trotting time, alternated with walking, every 2 weeks (up to 20 min of trot with 20 min of walking). However, the animal did not show any improvement at the clinical or ultrasonographic level.

**Figure 1:**
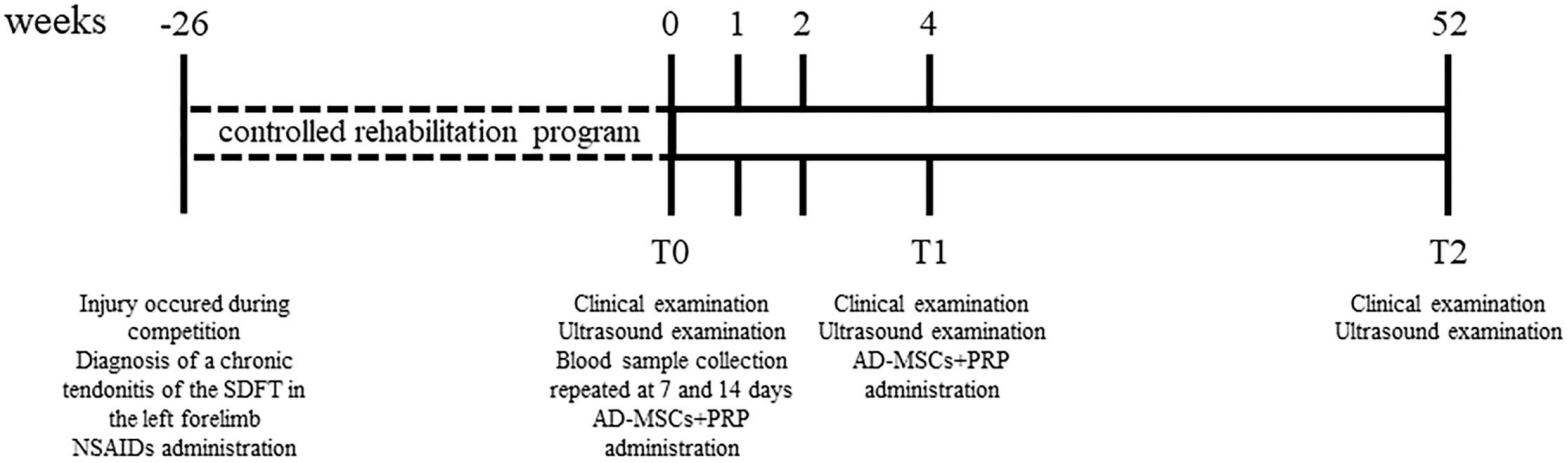
Schematic timeline of the clinical case reporting clinical examinations, ultrasonographic evaluations and therapy protocol.

In Figure 1 it is showed a schematic timeline of the clinical case.

### Diagnostic Imaging: Ultrasound Evaluation

Ultrasonographic evaluations of the central metacarpal region of both forelimbs were performed using a 7.5-MHz linear transductor probe. For each assessment, a complete examination of the SDFT was conducted by means of longitudinal and transverse scans. The obtained images were evaluated and scored (from 0 to 3) at each examination for two parameters as previously described (24, 39): lesion echogenicity and lesion longitudinal fiber alignment (FA). Criteria for scoring are listed in Table 1. The contralateral healthy limb was used as comparison.

The SDFT, in the middle third of the metacarpal region, presented with a focal hypo-echogenic area together with an irregular fiber alignment, which corresponded to ~ 30% of the cross-sectional area of the tendon. The lesion presented with a proximo-distal size of 36.4 mm.

### Treatment and Follow-Up

Treatment protocol consisted of ultrasound-guided intralesional injection with autologous adipose-derived MSCs (AD-MSCs) combined with PRP. AD-MSCs and PRP were isolated, characterized, and prepared for treatment as previously described (22, 40).

The adipose tissue was collected from the region above the dorsal gluteal muscle, at the base of the tail, because of the ease of access and absence of large veins. The horse was intravenously sedated with 0.01 mg/kg detomidine (Domodesan®, Orion Pharma, Italy); then the area was shaved, aseptically prepared, and locally anesthetized with 2% lidocaine (Lidor®, Richter Pharma AG, Italy). An incision of ~ 5–6 cm in length was made parallel 15 cm lateral to the spinal column, in order to allow visualization of adipose tissue between the skin and the musculature. Afterwards, ~ 4 g of subcutaneous adipose tissue was collected and stored in proper medium for transport, consisting of phosphate buffer saline (PBS) supplemented with penicillin-streptomycin (10%). Upon arrival to the laboratory, the sample was washed with PBS three times, minced and placed in a 0.01% collagenase type IA (Sigma-Aldrich, Italy) solution for 1 h at 37°C with continuous shaking. After digestion, the solution was filtered using a 100 μm cell strainer and diluted in DMEM high glucose (Sigma-Aldrich, Italy) supplemented with 10% FBS (Sigma-Aldrich, Italy). Afterwards, the solution was centrifuged twice at 300 xg for 10 min. Isolated cells were seeded in a culture flask in complete cell growth medium consisting of DMEM high glucose (Sigma-Aldrich, Italy), FBS 10% (Sigma-Aldrich, Italy), and 1% antibiotics (penicillin/streptomycin; Aurogene, Italy), maintained in culture, and expanded. Cells used for application were at passage 3 and 5. Isolated cells were characterized by flow cytometry and *in vitro* tri*lineage* differentiation as previously described by (40) and stated by (41).

The PRP was obtained by following a double centrifugation tube method in sterile conditions (first centrifuge at 1300 xg for 20 min and then at 300 xg for 15 min) as described by (42).

On the day of treatment, 10^7^ AD-MSCs were diluted in 4 mL of autologous PRP. Cell viability was assessed by trypan blue staining and more than 90% of cells were viable. Before injection, the horse was sedated with 0.01 mg/Kg detomidine (Domodesan®, Orion Pharma, Italy) and 0.1 mg/Kg butorphanol (Nargesic®, Acme Srl, Italy), and the injection area was aseptically prepared. The treatment was inoculated in the lesion site using a sterile 14G needle *via* ultrasonic guidance. Afterwards, protective sterile bandages were applied to the limb and the horse followed a rehabilitation protocol.

A second injection was performed 4 weeks later in order to continue the stimulation of the healing process. The applied protocol was the same as that used for the first treatment.

### Blood Plasma Analysis

Blood plasma was obtained after collection of peripheral blood from the jugular vein of the horse using a lithium-heparin sterile tube (BD Vacutainer®, BD, Italy) on the day of injection, and after 1 and 2 weeks post-treatment.

The blood plasma was analyzed for assessing levels of protein related to inflammation and the relative oxidative stress such as advance oxidation protein products (AOPP), carbonyl group (CT), malondialdehyde (lipid peroxidation *via* thiobarbituric acid reactive substance, TBARS), and the presence of thiols (thiol/disulfide reaction of thiol); all parameters were measured by following published protocols (43–46).

In addition, the concentration of proteins involved in the inflammatory process (IL-1β, MBS2020285, MyBioSource, US; IL-10, ab155466, Abcam, UK) or tendon wound healing (PDGF, MBS907132, MyBioSource, US; IGF-1, MBS7606417, MyBioSource, US) was estimated using enzyme-linked immunosorbent assay (ELISA) commercial kits specific for the equine specie. The analyses were performed following the manufacturer's protocol.

All samples were analyzed in triplicate in a 96-well plate and absorbance was obtained by using a VICTOR multilabel plate reader (Perkin Elmer, US).

## Outcomes

### Clinical Evaluation

Clinical assessments were performed every 2 weeks starting from the day of the first injection. In this study we report clinical outcomes on the day of injection (T0), at 4 weeks (T1) and 52 weeks (T2) after injection. Before treatment, the horse presented pain at palpation along with local heat and severe swelling in the mid-metacarpal region of the left forelimb. In addition, on the AAEP scoring system the horse showed a Grade 2.5/5 lameness.

At T1, swelling and pain of the injured region of the left forelimb were decreased; moreover, a reduction of lameness (Grade 1.5/5) was observed. The horse was able to load more weight on the affected limb. A second injection of the combined treatment was performed on the same day. After 1 year (T2), no signs of swelling, pain at palpation and lameness of the affected limb were observed. The horse presented with a Grade 0/5 lameness as it was able to trot fine under all tested circumstances. The horse showed a complete restoration of function and returned to sport activity; currently, the horse is still competing.

### Ultrasound Evaluation

**Figure 2:**
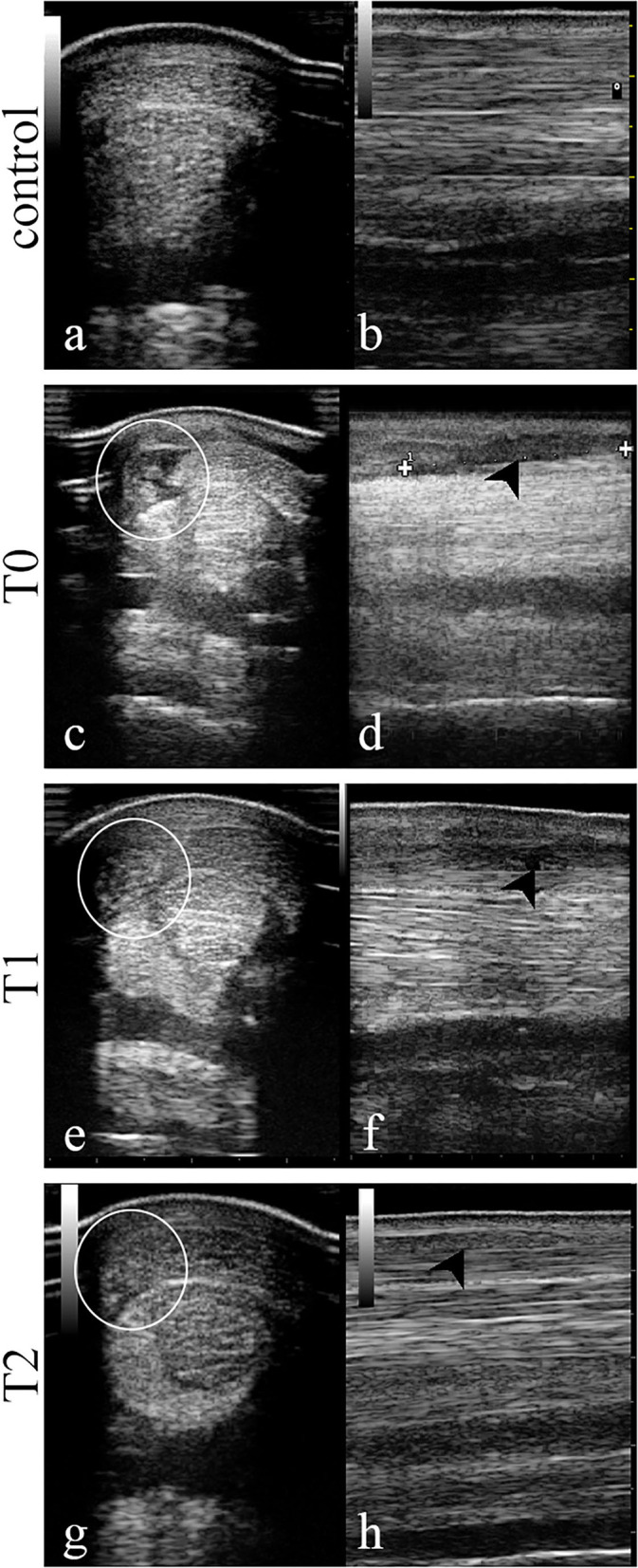
Transversal and longitudinal images of the central area of the superficial digital flexor tendon in the mid-metacarpal region performed at day 0 (T0, day of therapy injection), T1 (4 weeks), and T2 (52 weeks). At T0 the SDFT is characterized by a hypoechoic area **(c)** along with an abnormal fiber alignment **(d)**. After 4 weeks (T1), the same area resulted less hypoechoic **(e)** and the fiber pattern was more aligned **(f)** while at T2 the area showed a normal echogenicity **(g)** and fiber disposition **(h)** as in the sound contralateral limb **(a,b)**. White circle, corresponding injury area in transverse images; black arrow-head, injury area in longitudinal images.

Ultrasound examinations were assessed using the scoring system previously described (Figure 2). At T0, the tendon presented with a focal hypoechoic area (Grade 2.5/3) with a low FA (Grade 3/3). At T1, a slight increase in FA (Grade 2/3) was observed along with a small increase of echogenicity in the wounded area (Grade 2/3). At T2, 1 year after the first injection, echogenicity and fibers alignment were similar to the contralateral sound tendon (Grade 0/3).

### Blood Plasma Analysis

The concentration level of inflammatory and growth factors present in blood plasma was assessed on the day of injection, and after 1 and 2 weeks to evaluate markers involved in the tendon healing process (Table 2).

**Table 2:** Obtained data from analysis of blood plasma markers related to inflammation and tendon healing process.

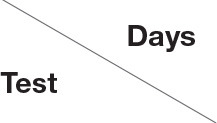	**0**	**7**	**14**	**Unit**
**AOPP**	0,77	0,70	0,43	nmol/mg
**TBARS** **(MDA)**	0,013	0,011	0,007	nmol/mg
**CT**	0,16	0,18	0,08	nmol/mg
**Thiols**	6,24	6,96	5,04	nmol/mg
**IL-1β**	0,084	0,085	0,069	ng/mL
**IL-10**	1,23	1,23	1,37	ng/mL
**IGF-1**	56,24	61,55	51,36	ng/mL
**PDGF**	3,12	14,50	6,43	ng/mL

The quantity of plasma oxidative stress products, as AOPP and TBARS, decreased gradually over time, with the lowest value at 14 days after the first treatment; CT and thiol groups showed a little increase at day 7, and then decreased at 14 days.

The pro-inflammatory cytokine IL-1β showed a decrease at 14 days while IL-10, an anti-inflammatory interleukin, showed an increase in its concentration on the same day.

The plasma concentration of growth factors, PDGF and IGF-1, showed an increase in their concentration at 7 days Post-treatment compared to the baseline values. After 14 days, a decrease of concentration of both factors was observed.

## Discussion

Superficial digital flexor tendon (SDFT) injuries are a severe problem that affect a large percentage of athletic and pleasure horses; often they develop recidivisms and, in the worst scenario, have to retire from competition early (9, 47). In a One Health perspective, the equine could play an important role as a model for human musculoskeletal disorders because of their similarities (48–50), especially for investigating the regenerative efficacies of innovative treatments such as PRP, bone marrow aspirate (BMA), or MSCs. However, in human medicine there is a limited knowledge about the combinatory application of these therapies, which is restricted to the treatment of knee osteoarthritis (51, 52) and rotator cuff rupture (53).

In the present case report, repeated ultrasound-guided intralesional injections of autologous AD-MSCs combined with autologous PRP were applied for the treatment of a recurrent SDFT lesion, that had naturally occurred in the left forelimb of a show jumping horse. The horse presented with a chronic tendonitis of the SDFT of the left forelimb occurred during sporting activity, which developed from a previous injury 6 months before the treatment herein described. The horse was unresponsive to conventional treatments such as NSAIDs and a controlled rehabilitation program. Therefore, the double application of AD-MSCs with PRP for the treatment of a naturally occurring lesion in the SDFT was the novel approach chosen.

Results were beneficial for the horse as the repeated injections of AD-MSCs and PRP resulted in a positive development of a SDFT chronic tendonitis over a 1-year follow-up. In addition, during this period the horse did not suffer any re-injury. The combined treatment might be accountable for the decrease of inflammatory markers (i.e., plasma protein levels) as observed during the first 2 weeks, which might have eventually led to the slight reduction of clinical symptoms observed at T1. The ultrasonographic evaluation showed restoration in structure, echogenicity, and fiber organization of the affected tendon after 1 year. This could also suggest that the biomechanical properties of the tendon were restored to an adequate level for allowing the horse to go back to the same performance status as before the injury. The addition of PRP to MSCs for the treatment of different disorders as skin wounds (54) or bone defects (55), has demonstrated to boost the regenerative effects of MSCs, both morphologically and functionally. The same effect was also observed for treating degenerative joint disorders in equine specie (56). In all models, the observed results were achieved principally by extracellular matrix remodeling, mainly dictated by the structural action of MSCs, which is a fundamental component in tendon healing. The underlying reason may be ascribed to the soluble molecules (e.g., growth factors) present in the PRP that might stimulate MSCs proliferation and release of bioactive factors (57). Indeed, MSCs themselves are able to release a plethora of soluble bioactive molecules that possess beneficial effects (58).

During the first 2 weeks after treatment, blood plasma analysis showed a reduced concentration of inflammatory markers (AOPP, TBARS, CT, and IL-1β) along with an increase of thiols at day 7 and a reduction at day 14. The substantial increase in the concentration of thiols after 1 week may be related to their function as antioxidants (59), while reducing at day 14 as the inflammatory process started to subside. Concomitantly, the concentration of IL-10, an anti-inflammatory cytokine, increased after 2 weeks. IL-10 is known to have a pro-mitotic effect on tenocytes and tendon-derived stem cells, stimulating cell proliferation and migration (60). In addition, the growth factors IGF-1 and PDGF showed an increase 1 week post-treatment. Both growth factors have positive effects on tendon cells, inducing proliferation plus attracting them to the wound area and stimulating ECM deposition (61, 62).

These observations were reflected 4 weeks after treatment (T1) by a reduction of pain and swelling of the affected forelimb area along with a partially reduced lameness. However, at the same time point, ultrasound images still presented with a hypoechoic area in the SDFT, corresponding to the diagnosed lesion. For this reason, the same treatment was applied a second time. The double application of AD-MSCs and PRP in a 30-day interval might have provided a prolonged activation of tendon regeneration due to a more protracted exposure, at the injury site, to both constituents. Moreover, the use of autologous sources for the therapy did not provoke any immune reactions to the horse after application, confirming the safety of MSCs (63).

To conclude, a repeated injection of autologous AD-MSCs coupled with PRP over a 52-week period supported a positive progression of a chronic SDFT lesion, which developed from a previous healed injury in a show jumping horse, allowing the animal to go back to competition. Probably, the measurement of inflammatory and oxidative stress markers in blood plasma can play a pivotal role in monitoring the healing process. However, these outcomes should be confirmed in the future by large placebo-controlled studies with animals affected by SDFT chronic tendonitis.

## Conclusion

To our knowledge this is the first case report of a successful treatment of a naturally occurring chronic SDFT tendonitis developed from a re-injury in a show jumping horse by a repeated and combined application of AD-MSCs with PRP. The therapy demonstrated to be safe and effective as no adverse reactions were observed; moreover, the horse was able to go back to competition. Our result might encourage the combined application of MSCs and PRP for the treatment of tendon injuries in equine clinical practice.

## Data Availability Statement

The original contributions presented in the study are included in the article/supplementary material, further inquiries can be directed to the corresponding author.

## Ethics Statement

Ethical review and approval was not required for the animal study because the owner of the horse signed a written consent, in which all treatment procedures were widely explained. All consent items were discussed in detail during the consultation visit to provide the owner with an overview regarding the benefits of the treatment and the expected results. Written informed consent was obtained from the owners for the participation of their animals in this study.

## Author Contributions

AC, FB, MP, and II followed the clinical case and performed the sample collection. LM, AC, LD, and AP performed cell processing and laboratory analysis. LM, AC, and NE prepared the manuscript. LM and MP contributed to study design and supervised it. GG and MP revised and edited the manuscript. All authors read and approved the final manuscript.

## Funding

This research was funded by Italian MIUR, Grant Number 2017FNZPNN (PRIN 2017, PI: MP).

## Conflict of Interest

The authors declare that the research was conducted in the absence of any commercial or financial relationships that could be construed as a potential conflict of interest.

## Publisher's Note

All claims expressed in this article are solely those of the authors and do not necessarily represent those of their affiliated organizations, or those of the publisher, the editors and the reviewers. Any product that may be evaluated in this article, or claim that may be made by its manufacturer, is not guaranteed or endorsed by the publisher.
